# Optimising Paediatric Transition to Intensive Care for Adults (OPTICAL): study protocol for a mixed method study

**DOI:** 10.1136/bmjopen-2025-101362

**Published:** 2025-07-16

**Authors:** Qi Huang, Charmaine Kohn, Sue Ben Abraham, Katherine Malbon, Anjalika Mallick, Paul R Mouncey, Kate Oulton, Christina Pagel, Louise Rose, Sarah E Seaton, Julie Taylor, Rum Thomas, Clare Windsor, Jo Wray, Padmanabhan Ramnarayan, Sonya Crowe

**Affiliations:** 1 University College London Clinical Operational Research Unit, London, UK; 2 Centre for Outcomes and Experience Research in Children’s Health, Illness and Disability, Great Ormond Street Hospital for Children, London, UK; 3 Patient and Public Involvement and Engagement (PPIE) Advisor and Representative, London, UK; 4 Paediatric Critical Care, Imperial College London Department of Surgery and Cancer, London, UK; 5 Department of Paediatrics, Imperial College Healthcare NHS Trust, London, UK; 6 Clinical Trials Unit, Intensive Care National Audit and Research Centre, London, UK; 7 King’s College London Florence Nightingale Faculty of Nursing Midwifery and Palliative Care, London, UK; 8 University of Leicester Department of Population Health Sciences, Leicester, UK; 9 Paediatric Critical Care, Sheffield Children’s Hospital NHS Foundation Trust, Sheffield, UK; 10 Rotherham NHS Foundation Trust, Rotherham, UK

**Keywords:** Adolescent, Paediatric intensive & critical care, Adult intensive & critical care

## Abstract

**Abstract:**

**Introduction:**

An increasing number of teenagers and young adults (TYA) with chronic conditions and complex needs are transitioning from paediatric to adult services, including admission to intensive care units (ICUs). As these services are often ill-equipped to care for TYA, there is a risk of compromised care. Despite recent guidelines from the UK Paediatric Critical Care and Intensive Care Societies highlighting the importance and urgency of improving ICU transition, current recommendations are not evidence-based and established pathways for ICU transition remain limited.

**Methods and analysis:**

This mixed-methods research study aims to generate evidence to underpin national policy on transition from paediatric to adult ICUs that will improve clinical care and patient experience. To do this, we will: (1) link and analyse UK national data (years 2017–2024) on paediatric and adult ICU admissions, hospital inpatient, outpatient and emergency care visits and survival status, to determine the clinical characteristics and healthcare resource utilisation from teenage years to early adulthood of people admitted to an ICU as a young person (admission aged 14 and 15), and how these relate to ICU admissions after age 16; (2) conduct semistructured interviews, online forums and surveys with TYA patients, carers and health professionals to understand their experience of transition in ICU services; and (3) synthesise these strands of evidence and use a structured process of stakeholder engagement to propose potential targeted improvements as appropriate.

**Ethics and dissemination:**

This study was approved by the East of England - Cambridge South Research Ethics Committee on 1 August 2024 (research ethics committee number 24/EE/0108), and the Health Research Authority Confidentiality Advisory Group (CAG) on 7 October 2024 (CAG number 24/CAG/0068). Study results will be actively disseminated through peer-reviewed journals, conference presentations and accessible lay texts and graphic summaries for the use of charities and patients including those with learning disabilities and neurodevelopmental disorders.

**STRENGTHS AND LIMITATIONS OF THIS STUDY**

- This is a national study that will examine the process of transitioning from paediatric to adult intensive care unit (ICU) across a whole population.
- This is a mixed methods study drawing on data linkage, qualitative interviews, online forums and quantitative surveys.
- We aim to interview a diverse range of participants, being as inclusive as possible, including those with learning disabilities and neurodevelopmental disorders.
- The study is limited by the relatively short time frame covered by the linked data set (2017–2024).
- The study is somewhat limited by the lack of clinical details in ICU admissions due to data minimisation in the paediatric ICU data set.

## Introduction

 Advances in medical care have extended life expectancy for children with conditions such as extreme prematurity, cystic fibrosis, neuromuscular disorder and congenital heart disease, leading to a growing need for adult healthcare services as they transition to adulthood.^1^,^3^ In this context, transition is defined as a ‘purposeful and planned process of supporting young people to move from children’s to adults’ services’.^4^ The need to improve healthcare transitions for teenagers and young adults (TYA) is well recognised and the subject of several reports, including guidelines from the National Institute for Health and Care Excellence.^4^,^8^ However, healthcare services remain inconsistent and inequitable in addressing this need, especially for conditions that include learning disabilities and neurodevelopmental disorders.^9 10^

In England, critically ill patients aged 16 years and older are generally cared for in adult intensive care units (AICUs), and those younger than 16 years are cared for in paediatric intensive care units (PICUs).^11^ While AICUs are available in most English hospitals, PICUs are centralised into only 28 hospitals, meaning that critically ill children presenting to hospitals without a PICU are transferred to a PICU in another hospital.^12^ Over the past 30 years, the pathology of patients requiring PICU admission has shifted from predominantly healthy children requiring short-term care for infection or trauma to an increasing proportion of children with medical complexity due to chronic conditions, including life-limiting conditions and technology dependence.^13 14^ Some require frequent, prolonged and repeated PICU admissions for recurrent acute health deteriorations.^15 16^ Many of these children are now surviving to an age beyond which they should be cared for in PICU, yet few strategies or established processes address their transition from paediatric to adult intensive care services, except for some specific conditions such as congenital heart disease.^17^

Differences in the organisation and practice of PICUs and AICUs, such as patient demographics, ICU environment, consent procedures and family support and experience, complicate transition. The principles of effective healthcare transition between paediatric and adult services, such as ensuring continuity, patient-centred care and coordinated planning, must be applied to critically ill TYA; however, achieving this remains a challenge due to the unpredictable nature of ICU transition, where the timing and location are often unknown, unpredictable and require the collaborative efforts of both paediatric and adult critical care teams. The UK PICU centralised structure often means TYA must access AICU in a different hospital to the PICU they have used in childhood, so information sharing and continuity of care can also be challenging. Although a high-quality transition (eg, advanced care plans, resuscitation and medical escalation status, airway and ventilation management) is crucial, especially for TYA with learning disabilities, PICU to AICU transition planning remains insufficiently addressed.^18^

This paper outlines a protocol of a national (England) mixed methods study that aims to build an evidence base to improve the care and experience of TYA transitioning from PICU to AICU services. We aim to (1) determine clinical characteristics and healthcare resource utilisation from teenage years to early adulthood of people admitted to ICU as a young person (age 14–15), and how these relate to ICU admission after the age of 16 years; (2) understand the experience of patients, family carers and healthcare professionals of transition from paediatric to adult ICU services, including barriers faced, examples of good developmentally appropriate practice and suggestions for improving the support provided; and (3) establish evidence-based potential improvements in the processes and support for transitioning to adult ICU services, targeted to specific patient groups as appropriate.

## Methods and analysis

### Study design

**Figure 1:**
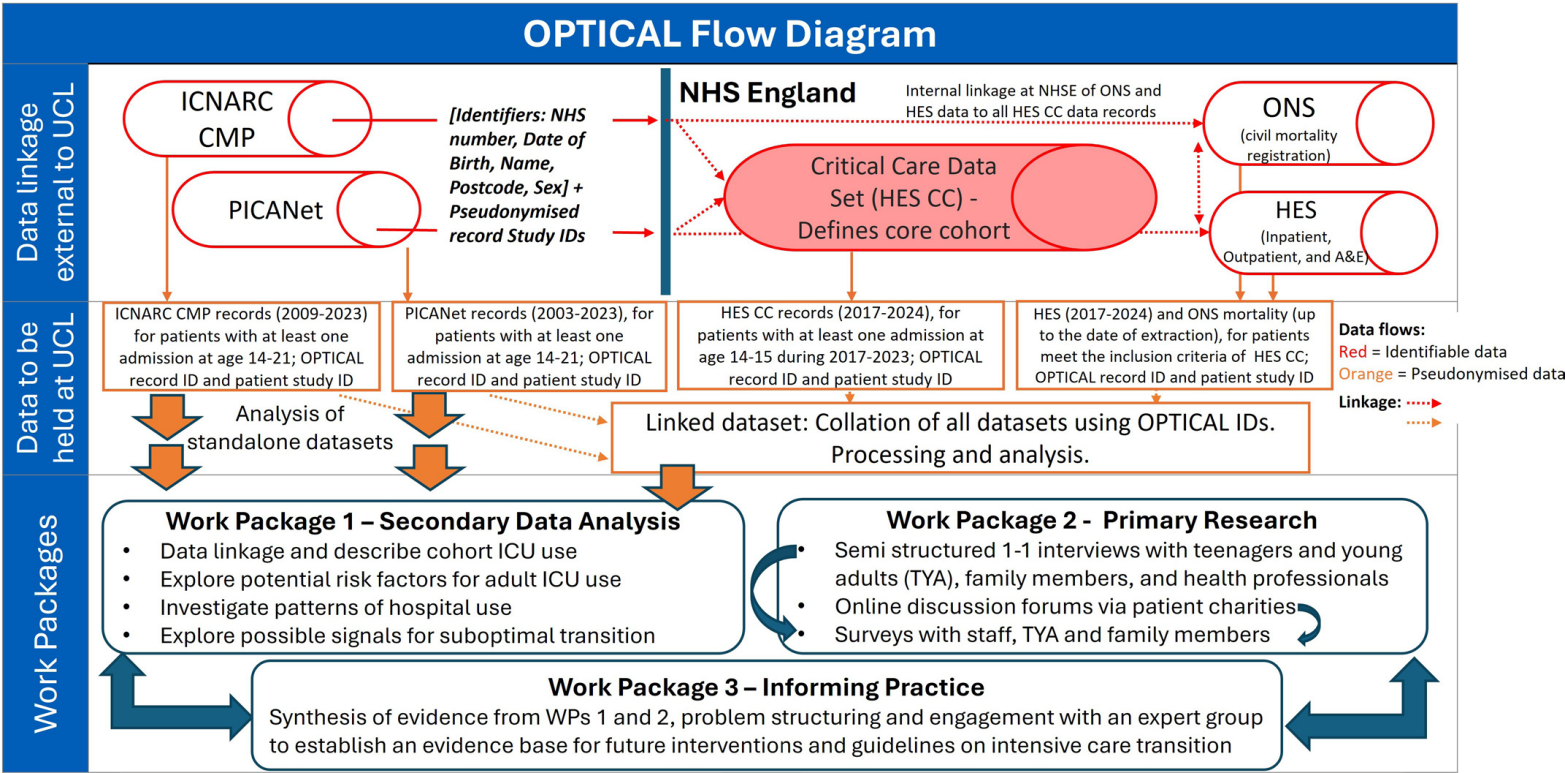
Data linkage and study flow diagram. The standalone data sets (PICANet and ICNARC CMP) cover a longer time period than the core data set HES CC and include more detailed clinical information, enabling analysis of ICU usage trends over time and patient pathways. A&E, accident and emergency; HES CC, Hospital Episode Statistics Critical Care; ICNARC CMP, Intensive Care National Audit and Research Centre Case Mix Programme; ICU, intensive care unit; NHS, National Health Service; NHSE, NHS England; ONS, Office for National Statistics; OPTICAL, Paediatric Transition to Intensive Care for Adults; PICANet, Paediatric Intensive Care Audit Network; UCL, University College London; WP, Work Package.

Optimising Paediatric Transition to Intensive Care for Adults (OPTICAL) is a mixed-methods research study with secondary data analysis (Work Package (WP) 1), primary qualitative research (WP2) and problem structuring/stakeholder engagement including synthesis of the findings from WPs 1 and 2 (WP3). Figure 1 provides information about the data flow and the mapping of the work packages together. The study began on 1 November 2023 with the expected end date of 31 July 2026.

### Work Package 1: data linkage and analysis

The objectives of WP1 are to determine the clinical characteristics and healthcare resource utilisation from teenage years to early adulthood of people who used intensive care aged 14 or 15, and how these relate to their intensive care use after the age of 16 years.

#### Data and sample size

There will be three data sets: two standalone national ICU audit data sets and a linked data set (which combines the core critical care data with the standalone data sets, hospital use data and survival status). Table 1 provides descriptions of all data sources, all of which are routinely collected clinical audit or national data sets, and the inclusion and exclusion criteria used to select patients from these data sets.

**Table 1:** Data sets, characteristics of interest and inclusion and exclusion criteria for the WP1 analysis

Data set name(Data controller)	Description	Key fields for analysis	Years data collected	Inclusion criteria	Exclusion criteria
Linked data set					
Hospital Episode Statistics (HES) Critical Care (HES CC) (NHS Digital) (core data set)	Critical care activity data collected for administrative purposes for every patient treated in England. Note that critical care can at times be delivered outside of an ICU.	Type of support received (paediatric activity codes and adult groupings) and duration, whether planned/unplanned admission.	Adult critical care since 2008Paediatric critical care since 2017	All patients in HES CC with at least one critical care admission in England aged 14–15 years between January 2017 (earliest HES CC data for children) and December 2023 (giving at least 1 year of follow-up). Data records will be available up to December 2024.	Any patient not represented in the Hospital Episode Statistics Critical Care (HES CC) data set with at least one critical care admission in England aged 14–15 years between January 2017 and December 2023.
HES inpatient admissions (HES APC), outpatient appointments (HES OP), A&E attendances (HES A&E) (NHS Digital) (linked to HES CC)	Administrative data from English hospitals, including demographics, diagnoses and healthcare utilisation.	Demographics, ICD-10 diagnosis codes, operation codes, health research groups (HRGs), length of stay.	APC since 1998OP since 2003A&E since 2007		
HES-ONS (NHS Digital)	Mortality data from the Office for National Statistics (ONS) linked to patient NHS number in HES, with every death registration in England.	Date of death, cause of death groupings (based on ICD-10 codes) and location of death	N/A—latest status data available for all those with an NHS number in England.		
Standalone data set (which will also be linked to the core data set HES CC for those who meet the inclusion criteria of the linked data set)					
Paediatric Intensive Care Audit network (PICANet) (HQIP)	High quality clinical audit with more clinical detail than HES on case mix, intensive care support and outcome, for all admissions to paediatric intensive care units (PICU) in England. Note that sometimes young adults are treated in PICU.	Whether emergency or elective, detailed diagnosis and comorbidity read codes, HRGs, interventions (type and duration).	Data since 2003	All records from data set in 2003 (have full coverage and good quality) to 2023 pertaining to patients who had at least one PICU admission between 14 and 21 years of age. Data records will be available up to December 2024.	Patients who did not have an ICU admission between the ages of 14 and 21 recorded in PICANet.
Intensive Care National Audit and Research Centre-Case Mix Programme (ICNARC-CMP) (ICNARC)	High quality clinical audit with information on clinical condition and adult intensive care units (AICU) support (beyond that captured in HES) for all admissions to general AICUs in England. Note that sometimes children are treated in AICUs.	Reasons for admission (ICNARC coding method), physiology and patient history (comorbidities), source of admission, whether planned/unplanned, service provision and duration, HRGs and SOFA, APACHE and other risk scores.	Data since 1994	All records from data set in 2009 (have full coverage and good quality) to 2023 pertaining to patients who had at least one AICU admission between 14 and 21 years of age. Data records will be available up to December 2024.	Patients who did not have an ICU admission between the ages of 14 and 21 recorded in ICNARC CMP audits.

A&E, accident and emergency; APACHE, Acute Physiology and Chronic Health Evaluation; HQIP, Healthcare Quality Improvement Partnership; HRG, Healthcare Resource Groups; ICD-10, International Classification of Diseases 10th revision; ICU, intensive care unit; NHS, National Health Service; SOFA, Sequential Organ Failure Assessment; WP1, Work Package 1.

##### Linked data set (core data set HES Critical Care)

The core data set comprises all patients in Hospital Episode Statistics Critical Care (HES CC)^19^ with at least one critical care admission in England aged 14–15 years between January 2017 (earliest HES CC data for children) and December 2023 (see figure 1). HES CC data will be available until December 2024, ensuring at least 1 year of follow-up for all patients regarding ICU admissions. We estimate that there are around 1000 intensive care admissions of teenagers aged 14–15 per year in England, giving ∼8000 admissions from 2017 to 2023 inclusive.

This core data set will be linked to all available records from the earliest available data date to 2024 in HES in-patient admissions, HES outpatient appointment, accident and emergency attendances along with latest survival status from Office for National Statistics linked to HES at data extraction date. It will also be linked to records in the two standalone ICU data sets (see details below) for those who had an admission aged 14–15 during 2017–2023 as these contain much more detailed data on reasons for ICU admission and support provided.

##### Standalone data sets

- *PICU Admissions (Paediatric Intensive Care Audit Network: PICANet*): we will identify all records from 2003 to 2023 for patients who had at least one PICU admission in England between 14 and 21 years of age (most will be aged 14–15). We estimate that there will be ∼15 000 TYA, with some having more than one admission recorded.
- *AICU Admissions (Intensive Care National Audit and Research Centre Case Mix Programme: ICNARC CMP*): all records from 2009 to 2023 for patients who had at least one AICU admission in England between 14 and 21 years of age (most will be after age 16). We estimate that there will be ∼60 000 TYA, with some having more than one admission recorded.

PICANet data minimisation policy is that all records for patients over 18 years without a PICANet event for more than 5 years are fully anonymised. Hence, it will not be fully linkable to other data sets. Therefore, to create a data set that links across paediatric and adult intensive care, as well as other hospital use data, HES CC will be used as the core dataset.^19^

### Data management

For the linked data set, we will combine overlapping events (inpatient stays, ICU stays, outpatient appointments and A&E attendances) across data sets into ‘spells of care’.^20^ The care spell time-series will be used to define a patient’s trajectory through the hospital system. The standalone PICANet and ICNARC data sets will be organised into patient level data sets showing a patient’s use over time of PICU and AICU, respectively.

### Main cohort and a comparator cohort

Using the linked data set, we will identify two groups: (1) a main cohort of patients who had both an ICU stay aged 14–15 and at least one additional ICU stay after age 16, and (2) a comparator cohort of patients with an ICU stay at ages 14–15 who do not have records of ICU use after the age of 16 and did not die before the age of 16.

The sample size of the main cohort is difficult to estimate because this is an understudied population, and determining this number is one of the key aims of OPTICAL. Our study is also limited by a potentially small sample size, given the relatively short time frame of the linked data set (2017–2024). However, within this period, we will have access to the entire national cohort, providing the largest possible sample available for this population.

### Outcomes

We are not testing a hypothesis in this research but rather using the linked data sets to describe ICU use and the transition from PICU to AICU services. The main outcome is to develop an evidence base of TYA healthcare utilisation and patient/family experience.

### Data analysis and statistical methods

Our analyses will be largely descriptive. We will report counts (proportions %) for categorical variables and median (IQR) for continuous variables.

#### Analysis for the linked data set

We will describe the number and characteristics of 14–15 year olds that transition from PICU to AICU (main cohort) and potential factors associated with ICU use after age 16 (entire linked data set), including reason for ICU admission, ICU usage patterns, underlying health conditions, weight, severity of illness markers, region, ethnicity and socioeconomic status (measured using the Index of Multiple Deprivation, which is the official measure of relative deprivation in small areas in England). We will also describe patterns of hospital use (inpatient, outpatient, A&E) before and after age 16, indicative of public healthcare costs (based on publicly available average national costs per critical care service, and Healthcare Resource Groups codes) and regional or demographic variations in this resource use (main and comparator data sets).

**Figure 2:**
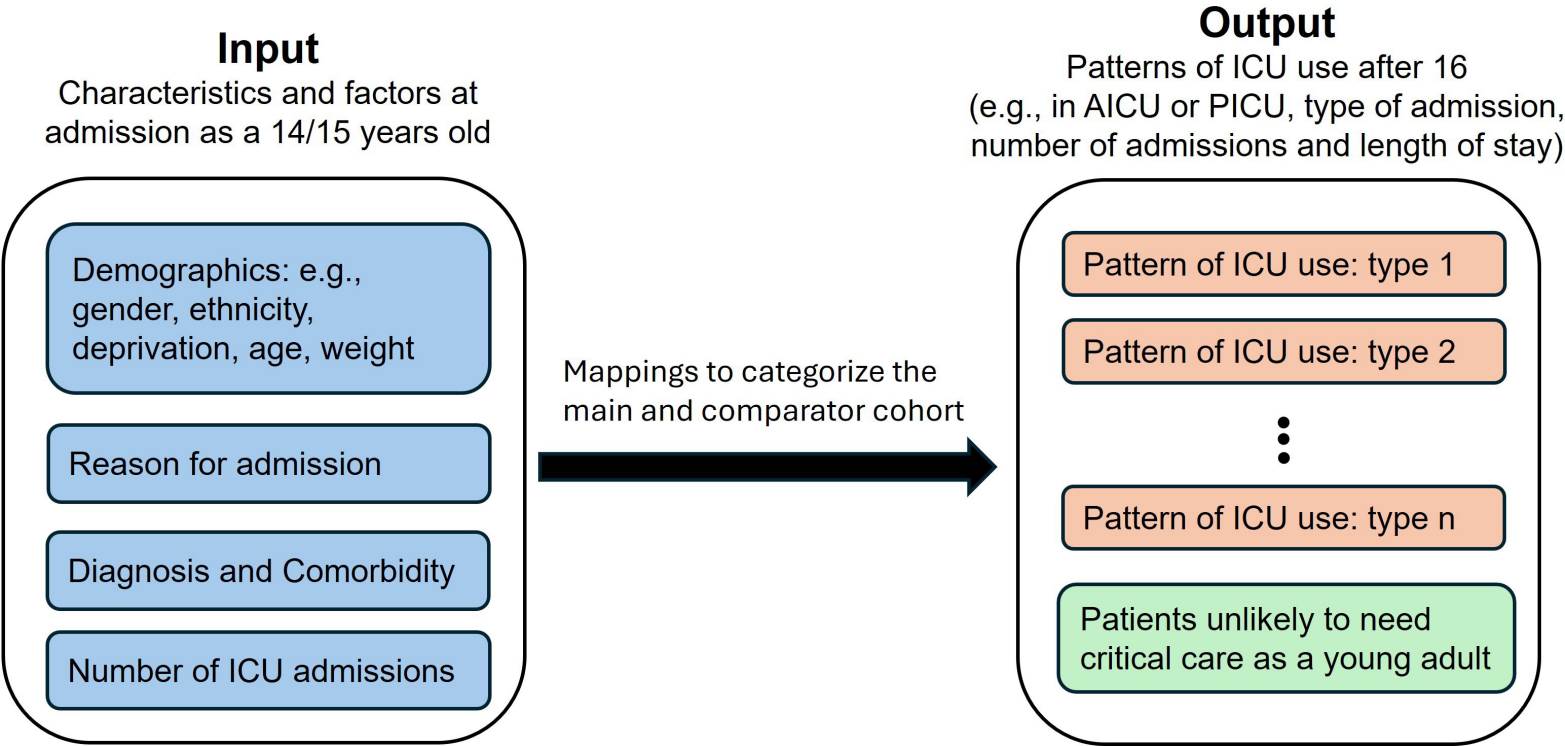
Schematic illustrating the classification of the main cohort (patients had at least one additional ICU stay after age 16) and those unlikely to require ICU care as a young person. AICU, adult intensive care unit; ICU, intensive care unit; PICU, paediatric intensive care unit.

We will develop mappings using available data on diagnosis, comorbidity, demographic characteristics and ICU usage patterns for 14–15 year olds to categorise patients into clinically meaningful, distinct patient groups with different patterns of ICU use after 16 (eg, number of admissions and lengths of stay), within the main and comparator patient cohorts (figure 2). Initial mapping will be developed using the linked data set to ensure that we capture the transitioning population, which will then be checked and refined on the larger standalone data sets.

#### Analysis for the standalone data set

Within the standalone data sets, it will not be possible to identify patients who transitioned from PICU to AICU outside of the subset of linked patients. Using the PICANet standalone data set, we will adapt categories developed using the linked data set (figure 2) to assign patients to groups who are likely to require intensive care aged 16 or over and those who are less likely to need another ICU admission. We will explore and compare patterns of PICU use between the assigned patient groups, to explore if we can identify those patients likely to need ICU in early adulthood. We will also examine temporal trends in the number of PICU patients likely to transition to AICU, enabling assessments of how demand for AICU resource and transition support might grow in the future.

Using the ICNARC CMP standalone data set, we will adapt our categories (figure 2) to assign patients to groups who were likely to have required intensive care at age 14 or 15, and those who were more likely to have been admitted to an AICU. We will explore and compare patterns of AICU use among the assigned groups, including AICU admission after the age of 21, to improve our understanding of future resource use of TYA in adulthood. We will also produce an updated description of AICU use by children aged <16 years.^21^

#### Statistical methods

Statistical methods will include multinomial logistic regression or multioutput decision trees (eg, random decision forests) analyses for developing the mappings of patients to ICU use patterns after age 16 based on reasons and diagnosis for admission, demographic factors and ICU use at ages 14–15. Factors potentially associated with ICU admission after 16 in those TYAs with an ICU admission aged 14–15 will be explored using competing risk analysis methods (cumulative incidence and cause-specific hazard models),^22^ accounting for the occurrence of competing event (death before age of 16). Hospital resource utilisation data will be reported per successive year of follow-up. Their association with risk factors will be evaluated by quantile regression.

### Missing data

At this stage, we are unaware of the level of missing data. In the linked data set, we will use data from different sources to enhance data completeness. For a high missing data rate (greater than 10%), we will investigate potential causes and examine case mix and outcomes among the missing data. If we believe that data are missing at random, we will apply multiple imputation; otherwise, we will conduct a complete-case analysis. Sensitivity analyses (eg, worst case analysis) will be conducted to investigate any assumptions. We will develop robust protocols for addressing missing data and inconsistencies once the levels are known and present these proposals to the study management group for review and approval before analysis begins.

### Work Package 2: qualitative interviews, online forums and quantitative surveys

WP2 aims to address the secondary outcome of understanding the experience of patients, carers and health professionals of PICU to AICU transition, including barriers they face, examples of good transition practices and suggestions for improving support and practice. This is a mixed-method workstream involving semistructured interviews, online forums and surveys to aid our understanding of ICU transition.

#### Semistructured Interviews

##### Study population

Semistructured interviews will target three key participant groups to gain comprehensive insights into the transition from PICU to AICU. These groups include: (1) healthcare professionals providing care to TYA in AICUs, or working in paediatric specialties, or community settings that prepare TYA for transition; (2) TYA aged 16–21 years who have had at least one PICU admission after age 14 and one AICU admission; and (3) family members or carers of these TYA. Approximately 120 interviews are planned, aiming for an estimated 35–40 interviews from each group. Such a sample size aims to facilitate adequate ‘information power’ by gathering sufficient information about the experience of ICU transition from multiple diverse groups, allowing for meaningful comparison within and across subgroups (eg, healthcare professionals vs TYA vs families/carers; demographics, clinical diagnosis). By ensuring representation across these groups, the study seeks to capture a comprehensive understanding of the challenges, facilitators and lived experiences involved in the transition process. Inclusion and exclusion criteria for all participant groups are summarised in table 2.

**Table 2:** Inclusion and exclusion criteria across the WP2 component of the OPTICAL study

WP2 component	Inclusion criteria	Exclusion criteria
Semistructured interview	Healthcare professionals caring for TYA in AICU, paediatric or community settings. TYA aged 16–21 with at least one PICU admission after age 14 and one AICU admission. Family members/carers of eligible TYA, including those who are bereaved or those who declined transition.	Individuals unable to provide informed consent.
Online forum	TYA who have had a PICU admission (with or without AICU transition). Family members or carers of TYA with PICU experience.	Participants who do not provide demographic information for eligibility confirmation.
Survey completion	Healthcare professionals in AICUs or paediatric centres preparing TYA for transition. TYA aged 16–21 with at least one PICU admission after age 14 and one AICU admission. Family members of TYA meeting the above criteria.	Surveys submitted without sufficient demographic information to confirm eligibility.

AICU, adult intensive care unit; OPTICAL, Paediatric Transition to Intensive Care for Adults; PICU, paediatric intensive care unit; TYA, teenagers and young adults; WP2, Work Package 2.

##### Eligibility criteria

Healthcare professionals eligible for inclusion are those working in relevant clinical or community roles, including nurses, doctors, allied health professionals, mental health workers and transition coordinators. Participants will be purposively sampled based on seniority, profession and specialty to ensure diverse representation. Sampling criteria for TYA participants include the nature of their AICU admission (elective or emergent), health conditions, age group (16–18 or 19–21 years) and specific needs, such as learning disabilities or autism. Bereaved family members or carers, or those whose TYA chose not to transition to adult ICU, will be eligible to participate, even if their associated TYA are not enrolled. Non-English-speaking participants will be included, with interpreters facilitating interviews to ensure inclusivity. Individuals unable to provide informed consent will be excluded.

##### Recruitment and consent

Recruitment will be conducted through Participant Identification Centres (PICs) located in 15–20 AICUs and 3–5 paediatric centres. These centres will be selected for diversity in size, location, specialisation and whether the hospital has a PICU as well as an AICU. Healthcare professionals will be contacted via emails distributed by PICs, which include study details and a consent-to-contact form. TYA and family members will be identified through recent AICU admissions, with clinical teams providing the study poster and a consent-to-contact form. Paediatric and community teams will identify those who declined transition or are on palliative care pathways, also providing a study poster and consent-to-contact form. Bereaved families will be approached sensitively through bereavement teams, adhering to a 1 year post-bereavement period.

Tailored participant information sheets (PIS) and consent forms will be provided to each group, explaining the purpose, scope and ethical approval of the study. Easy-read versions of the PIS and consent forms will be available for TYA with learning disabilities or other accessibility needs. Furthermore, to ensure accessibility and inclusivity of non-English speaking participants, all study materials, including PIS and consent forms, will be translated into relevant languages. Where the research team is informed in advance—either by NHS PICs or via direct requests from potential participants during the expression of interest process—translated materials will be prepared and provided prior to contact. Translation of documents, including checks for accuracy, and translators will be provided by existing translation services associated with NHS clinical services. Participants will be given adequate time to review the PIS and discuss it with researchers before providing consent. Informed consent will be obtained immediately prior to the interview, with interpreters available to facilitate the process when required, allowing participants to ask any questions they may have prior to giving formal consent. If used, interpreters will either attend in person (for face-to-face interviews) or join remotely for virtual interviews. Written consent will be obtained for face-to-face interviews, while verbal consent will be used for virtual or telephone interviews. In the case of verbal consent, the interviewer will read out each consent statement and the participant will be asked to verbally confirm their agreement. A paper copy of the completed consent form will be provided to the participant and stored by the research team. The researcher will reassure participants about their right to withdraw at any stage and provide information about the secure handling and anonymisation of their data.

While it is unlikely, we acknowledge that interpreter availability for all languages may not always be possible. In such cases, only participants for whom both translated materials and interpreter support can be provided will be included.

##### Data collection and analysis

Interviews will be conducted face-to-face for TYA and family members, where rapport-building is particularly important, and via telephone or video conferencing for healthcare professionals, offering flexibility. Each interview is likely to last between 45 and 90 min, guided by topic frameworks informed by transition literature and input from the study’s patient and public involvement (PPI) group. Topics will include barriers to transition, examples of good practice and changes in care delivery (such as shifts from family-centred to person-centred care). Topic guides will be iteratively refined based on early interviews to ensure alignment with participant perspectives. Demographic data will be collected to contextualise findings. All interviews will be audio recorded and transcribed by an independent translation service. Where a translator is required, the audio recording will be transcribed in both English and the language used by the participant and interpreter. Data from interviews will be analysed using the framework method, following Gale *et al*’s seven-stages of analysis; transcription, familiarisation, coding, development of a working analytical framework, indexing, charting and interpreting the data.^23^ Data sufficiency will be assessed through ongoing team discussion and be determined by consensus when thematic saturation has been reached in relation to the research question.

### Online forums

#### Study population

Online asynchronous forums provide an opportunity to gather experiences and perspectives from a broader group of participants, including those who may not participate in interviews. The forums will involve TYA who have been admitted to PICU and their family members or carers. Two forums, one for TYA and one for family members, are planned, involving approximately 200 participants. Inclusion and exclusion criteria are summarised in table 2.

#### Eligibility criteria

Eligibility is based on demographic information provided during registration. TYA are eligible to participate if they are aged 16 years and over and have relevant lived experience of being admitted onto a PICU, with or without a subsequent AICU admission. Family members or carers of a TYA who has been admitted onto a PICU are also eligible to participate within the forum. This will ensure that participants with diverse perspectives are represented.

#### Recruitment and consent

Recruitment will be facilitated by three partner charities, which will advertise the forums on their websites, newsletters and social media platforms. Interested participants will be directed to closed Facebook groups, where they will be asked to provide basic demographic information and respond to brief screening questions during registration for eligibility verification; however, full verification of identity will not be possible in an online context. On confirmation, participants will be able to join the asynchronous forums and contribute to discussions. Participation will be voluntary, and engagement with the forum will imply consent. Forum contributions will be monitored closely, and any responses that appear inauthentic or inconsistent with the study aims will be reviewed and, if necessary, removed.

A specific PIS tailored to the online forums will be available on charity websites. It will outline the study’s purpose, data anonymisation, moderation protocols and participants’ rights. Participants will be assured that their posts will be anonymised before analysis and that they can withdraw at any time while the forum is running, and their data will be withdrawn on request.

#### Data collection and analysis

Forums will be moderated by one of the partner charities who are experienced in moderating such discussions. Forums will run asynchronously over a 3-month period, allowing participants to contribute at their convenience. The charities will be responsible for posting prompts and new questions depending on participant responses as well as ensuring appropriate online behaviour. Prompts and questions will be developed during codesign workshops involving the research team, parent co-applicant and PPI group. Topics will include transition experiences, examples of good practice, barriers, differences in model of care, unexpected challenges and suggestions for improvement. Proven approaches to online moderation ensure respectful and constructive discussions.^24^,^26^ While data saturation will not be formally assessed, new prompts will be introduced pragmatically when engagement with a topic diminishes. Transcripts will be anonymised by the charities and participant responses will be analysed by the research team using reflexive thematic analysis following the approach developed by Braun and Clarke.^27 28^ This method allows for the identification and interpretation of meaningful patterns across the experiences shared while recognising the researcher’s active role in shaping the analysis through reflective engagement with the material.

### Survey completion

#### Study population

We will use surveys to provide broader, quantitative insights into transition experiences, capturing barriers, challenges and examples of good practice. We will recruit healthcare professionals involved in AICU transitions, TYA aged 16–21 years who meet the inclusion criteria, and their family members. Approximately 300 survey responses are anticipated in total. Inclusion and exclusion criteria are summarised in table 2.

#### Eligibility criteria

Eligibility criteria are the same as participants invited to interview. Demographic details will be collected during the survey to confirm eligibility.

#### Recruitment and consent

Surveys will be distributed widely through professional organisations, partner charities and social media. QR codes linking to the surveys will be widely shared and displayed on partner websites. Snowballing will be encouraged to expand participation. Accessibility will be prioritised, with easy-read versions and audio formats available.

Consent will be implied through survey submission. A tailored PIS will be provided via the survey link, detailing its purpose, scope, anonymity and voluntary nature. Participants will be informed of their right to withdraw before submission and reassured about secure data handling.

#### Data collection

Hosted on SmartSurvey, the surveys will include Likert-scale questions, forced-choice options and free-text responses, combining quantitative and qualitative data. Questions will be informed by findings from the interviews and forums, as well as PPI group input, addressing topics such as transition barriers, good practices and recommendations. Thematic analysis will be applied to free-text responses, while descriptive analysis will characterise trends in demographic and quantitative data.

### Work Package 3: informing practice

WP3 aims to establish the evidence base for future interventions and guidelines for ICU transition services. It does not involve a study population or recruitment and will be informed by and run alongside WPs 1 and 2 as an overarching strand of the project. Using the approach of Crowe *et al*,^29^ we will integrate the findings from the interviews, forums and surveys in WP2 and use these in combination with the quantitative data findings from WP1 in a structured stakeholder process to establish evidence-based potential improvements in the processes and support for transition from PICU to AICU services, targeted among patient groups as appropriate.

#### Stakeholder engagement

An expert group will be established to review evidence relating to the management of ICU transition, with representatives from four stakeholder groups:

- Children and teenagers who have been through PICU and AICU, and their carers.
- Bereaved parents/carers of TYA.
- Our partner charity organisations.
- Healthcare professionals (across AICU and PICU, ensuring representation of nurses, AHPs and doctors).
- Commissioners (from integrated care boards (which are responsible for AICUs) and NHS England (responsible for PICUs).

#### Systematic evidence synthesis

Findings from WP2 will be synthesised using the approach of Crowe *et al*.^29^ First, a ‘hyper-framework’ will be created by combining data from each of the separate frameworks generated from the interviews (with healthcare professionals, young adults and families), online forums (for young adults and families) and surveys (with healthcare professionals, young adults and families). In the hyper-framework, similar themes across the different originating sources, viewed from different perspectives (like family or healthcare professionals), will be merged into ‘hyper-themes’ and organised to reflect the TYA journey. Within each hyper-theme, data will be categorised as identifying a service issue, suggesting a service improvement (such as good practices), or as unrelated, with unrelated data removed. Common service issues will be grouped into ‘archetypal service problems’, each described from various perspectives. Candidate service improvements directly addressing these problems will be added, creating a set of potential improvements linked to specific issues.

#### Formal problem structuring methods

Operational research problem structuring methods (approaches used to tackle complex problems, focusing on understanding and structuring the problem before potentially applying mathematical techniques, eg, soft systems methodology)^30^ will be used to acknowledge and engage multiple perspectives in systematically considering potential changes to transition services, incorporating and integrating relevant evidence generated in the study in WPs 1 and 2. Activity will be centred on five workshops with the expert group:

- Workshop 1: Present study overview (aims, workstreams, timelines); Review emerging insights from the WP2 interviews; Review plans for identifying patient groups in WP1.
- Workshop 2: Review findings from WP2 interviews, in particular examples of best practice and suggestions that address identified service problems/gaps in support; Review draft questions for WP2 online forums; Review preliminary patient groups identified in WP1 for any prioritisation or customisation of support in transition.
- Workshop 3: Finalise the characterisation of patient groups that might benefit from different types of support or prioritisation in transition services (drawing on WP1 analysis); Review findings from WP2 online forums; Review draft survey questions and recruitment plans (WP2) in light of the finalised patient groups and learning from the interviews and forums.
- Workshop 4: Review findings from the surveys (WP2); Review findings from the quantitative data analysis (WP1); Use the final findings from WP1 and WP2 to establish initial evidence-informed potential improvements to the transition process.
- Workshop 5: Assess the feasibility and acceptability of each potential improvement identified in the study and any customisation or prioritisation for certain patient groups given their particular needs and any resource constraints; Finalise for wider endorsement (via our dissemination workshop, see below).

### Patient and public involvement

This study is rooted in powerful testimony from TYA and their families on the challenges of transitioning to AICU and the lack of preparation or pathway for TYA who will require AICU (also highlighted by our partner charities).

The PPI co-applicant has a defined and funded role within the research team, including reviewing the grant application and study materials, providing input on data collection methods and interpretation as well as leading the PPIE group. They will chair a PPI group with 2–3 TYA, 2–3 family members and one 2–3 charity representative. They will meet 2–3 times per year. Each PPI member will contribute 3.5 days over the course of the project. We will hold a 1 day face-to-face event, cofacilitated by our parent co-applicant, at which they will coproduce, in consultation with the wider research team, patient information and topic guides, surveys and questions for the discussion forums and ensure that the project remains patient and family focused, and accessible to all. Participants involved in the group will be reimbursed for their time in line with national guidance, in recognition of the valuable contribution they make to the research. Members of the PPI group will be involved in the dissemination of findings to patients and families and be offered the opportunity to contribute to journal publications, conference presentations and other dissemination activities. We aim to recruit at least one TYA with a learning disability who will be individually supported by a consultant nurse in learning disabilities. Terms of reference will be established for the group.

### Ethics and dissemination

The study was approved by the East of England—Cambridge South Research Ethics Committee on 1 August 2024 (research ethics committee number 24/EE/0108), and the Health Research Authority Confidentiality Advisory Group (CAG) on 7 October 2024 (CAG number 24/CAG/0068).

All quantitative data will be stored in the ISO27001 certified University College London (UCL) Data Safe Haven, which conforms to the NHS Information Governance Toolkit. Interviews will be transcribed by a trusted third party (TakeNote) and password protected anonymised transcripts will be stored on a secure server at Great Ormond Street Hospital (GOSH). Charities will anonymise the online forum transcripts before sending them to the research team for storage on the GOSH server. No identifiable information will be collected in the surveys, data from which will be stored on the GOSH server. We will comply with UK General Data Protection Regulation (GDPR) and UCL’s data confidentiality procedures.

We will produce a variety of outputs, such as academic peer-reviewed articles (results of WP1 will be reported in accordance with the STROBE (Strengthening the Reporting of Observational Studies in Epidemiology) guideline), conference presentations, webinars to present study findings (eg, through the professional societies and Royal Colleges), one-page executive summaries and accessible lay text/graphical summary (we aim to ensure accessibility for individuals who are non-English speakers or have learning disabilities or neurodevelopmental disorders). Our multiprofessional team will leverage their roles within professional societies to influence updates to the UK Intensive Care Society and UK Paediatric Critical Care Society transition guidelines, improve quality standards for transition care and enhance patient experience measures. We also aim to report key findings and recommendations to commissioners (integrated care boards and NHS England). Additionally, insights from testbed regions and partnerships with national clinical audits may shape future standards and metrics for effective PICU to AICU transitions.
