# Supplementary material for: Optimising Paediatric Transition to Intensive Care for Adults (OPTICAL): study protocol for a mixed method study
Source: BMJ Open. 2025 Jul 16;15(7):e101362. doi: 10.1136/bmjopen-2025-101362 (PMC12273106; doi:10.1136/bmjopen-2025-101362)
Supplement: Supplementary file 1 [file bmjopen-2025-101362.reviewer_comments.pdf]

PEER REVIEW HISTORY

BMJ Open publishes all reviews undertaken for accepted manuscripts. Reviewers are asked to complete a checklist review form and are provided with free text boxes to elaborate on their assessment. These free text comments are reproduced below.

ARTICLE DETAILS

Title (Provisional)

Optimising Paediatric Transition to Intensive Care for Adults (OPTICAL): Study protocol for a mixed method study

Authors

Huang, Qi; Kohn, Charmaine; Abraham, Sue Ben; Malbon , Katherine; Mallick , Anjalika; Mouncey, Paul R.; Oulton, Kate; Pagel, Christina; Rose, Louise; Seaton, Sarah E; Taylor, Julie; Thomas , Rum; Windsor , Clare; Wray, Jo; Ramnarayan, Padmanabhan; Crowe, Sonya

---

VERSION 1 - REVIEW

**Reviewer**	**1**
**Name**	**Perry-Eaddy, Mallory**
**Affiliation**	**University of Connecticut School of Nursing**
**Date**	**28-May-2025**
**COI**	**None**

I appreciate the opportunity to review this extremely important protocol for transition from pediatric to adult ICU care using a mixed methods approach. I have some suggestions below that may strengthen the manuscript.

- Line 11 – what is meant by “Intensive care is…low-volume”? To my understanding, PICU/AICU is highly specialized with large volumes each year, which is suggested by your datasets and estimates. Can you rephrase this?

- As described, I find the organization of the methods to be quite confusing. The inclusion of standalone datasets and the linked dataset is not completely clear, especially considering there is a lot of emphasis on how the standalone datasets will be incomplete. Line 76 states “hence it will not be fully linkable to other datasets.” If this is the case, can you clarify why these are needed in addition to the core dataset? This is also repeated in lines 141-142, “Of note, the mapping will not be perfectly applied in PICANet and ICNARC, because the standalone dataset does not contain all the fields of the linked dataset. The visual in Figure 1 doesn’t make it very clear.

- Within both the dataset sections there are estimates (i.e. lines 69-70; 73-74; 90-91). It would be helpful to understand how the author's surmised these estimates and what basis they are grounded. Also, a plan if the estimates are much lower than expected.

- There is often discussion of linked datasets out of context of the core dataset which I think adds to the confusion. For example, lines 63-4 reference "two standalone national ICU audit datasets and a linked dataset." However, line 104 describes linked datasets in a way that seems to combine all three Is this an analytic dataset that is being referenced?

- Additionally, restructuring the analysis plans may be useful, to the above point. Having the standalone first, followed by the "linked dataset" or vice versa, though remaining consistent in how they were introduced in lines 66-89.

- Lines 153-154 suggest missing data will be inferred from different sources. Which different sources?

- It is novel to include non-English speaking individuals; however, can you go into additional detail on how they will be consented? The methods discuss translators during interviews, though will short forms and/or translated consents be used? Are all of those languages available for consent? Also will translation be done by a person (limited language capabilities) or a virtual service (i.e. language line)?

- Line 221 describes using "the seven-stage process of transcription." Yet there is no citation to the methods for this transcription or the qualitative methodology which is mentioned later, "Framework" and thematic analysis.

- Individuals will be eligible for inclusion based on demographic information during registration. How will the authors ensure that individuals are actually within the target population of interest? (i.e. not an impersonator causing nonsensical responses)

- I commend the authors on inclusion of the PPI in study design. Is there compensation for this research? It seems that a considerable amount of time will be taken to participate in focus groups.

- There is mention of parent co-applicant. Can you expand on this? Is this for funding of this project, and if so, is it funded?

:

- Figure 1 is a bit confusing and doesn't answer as to why the standalone datasets are needed, and what exactly they are adding to the core dataset. Perhaps more in-depth explanation in the text, and figure legends may assist as figures should be able to stand on their own.

- Figure 2 outlines the trichotomous outcomes of ICU use in "Output." This needs to be expanded upon and described. What is meant by patterns, and what are the 3 types?

- There is little discussion regarding limitations besides small sample size. It is difficult to ascertain the true proposed sample size. Please specify.

- An additional limitation would be the inability to fully link the datasets into an analytic dataset due to differing variables.

**Reviewer**	**2**
**Name**	**Wu, Haiying**
**Affiliation**	**The First Affiliated Hospital of Kunming Medical University, Emergency Department**
**Date**	**05-Jun-2025**
**COI**	**None**

1.Clarify Integration Strategy Across Work Packages

Please elaborate more clearly on how findings from WP1 (quantitative data), WP2 (qualitative interviews and forums), and WP3 (stakeholder engagement) will be integrated. Consider including a schematic or paragraph detailing your planned triangulation or synthesis strategy.

2.Define Technical Terms

Some methodological terms such as "hyper-framework" and "problem structuring methods" may be unfamiliar to a broad medical audience. We recommend briefly defining these upon first use to improve clarity and accessibility.

3.Address Potential Sample Size Limitations

Although the study acknowledges a limited number of transition cases in the linked dataset (2017–2023), a more detailed discussion of how this may impact generalisability and statistical power would strengthen the protocol.

4.Provide Saturation Strategy for Qualitative Components

While you plan 120 interviews and 200 forum participants, please indicate how thematic saturation will be assessed and whether you anticipate iterative refinement based on early data.

5.Refer to Reporting Guidelines Explicitly

Consider including a brief note stating that the protocol follows relevant reporting standards (e.g., STROBE, SPIRIT, or GRAMMS for mixed-methods studies), and ensure that the checklist is submitted as supplementary material.

6.Consider Streamlining Background Section

The introduction is comprehensive but somewhat lengthy. Consider condensing the background by summarising widely known guidelines or prior studies into fewer lines to improve readability.

VERSION 1 - AUTHOR RESPONSE

Reviewer: 1
Dr. Mallory Perry-Eaddy, University of Connecticut School of Nursing, University of Connecticut School of Medicine

Comments to the Author:

I appreciate the opportunity to review this extremely important protocol for transition from pediatric to adult ICU care using a mixed methods approach. I have some suggestions below that may strengthen the manuscript.

Response:

Thank you for your comments.

Introduction
- Line 11 – what is meant by "Intensive care is…low-volume"? To my understanding, PICU/AICU is highly specialized with large volumes each year, which is suggested by your datasets and estimates. Can you rephrase this?

Response:

We used the phrase 'low volume' in comparison to general hospital admissions. However, we realise that this could cause confusion and misunderstanding, so we have removed it in the revised manuscript.

Methods
- As described, I find the organization of the methods to be quite confusing. The inclusion of standalone datasets and the linked dataset is not completely clear, especially considering there is a lot of emphasis on how the standalone datasets will be incomplete. Line 76 states "hence it will not be fully linkable to other datasets." If this is the case, can you clarify why these are needed in addition to the core dataset? This is also repeated in lines 141-142, "Of note, the mapping will not be perfectly applied in PICANet and ICNARC, because the standalone dataset does not contain all the fields of the linked dataset. The visual in Figure 1 doesn't make it very clear.

Response:

Sorry for the confusion. These standalone datasets will provide public benefit additional to the linked dataset as the data began several years earlier than the core dataset (HES critical care), allowing a more detailed analysis of ICU use over time and trends in growth of different cohorts of children who use ICU frequently. They also include more detailed clinical information, such as primary diagnoses, comorbidities, reasons for admission, and treatments received. This additional detail

helps us better understand patient pathways and develop risk factors when linked to the core dataset.

Ideally, the standalone PICANet data should serve as the core dataset. However, due to its data minimisation policy, it cannot be fully linked to other data, such as adult ICU and hospital use.

In the revised version, we have highlighted the advantages of standalone dataset in line 72-73, and added one sentence in the Figure 1 legend to explain the importance of including them:

"The standalone datasets (PICANet and ICNARC CMP) cover a longer time period than HES CC and include more detailed clinical information, enabling analysis of ICU usage trends over time and patient pathways."

The sentence "Of note, the mapping will not be perfectly applied in PICANet and ICNARC, because the standalone dataset does not contain all the fields of the linked dataset" was misleading. What we meant to say was that the standalone dataset does not contain all the admissions from the linked dataset. Since this point is already stated in the first paragraph under the section "Analysis for the standalone dataset," we have deleted the sentence in the revised version.

- Within both the dataset sections there are estimates (i.e. lines 69-70; 73-74; 90-91). It would be helpful to understand how the author's surmised these estimates and what basis they are grounded. Also, a plan if the estimates are much lower than expected.

Response:

Those estimates were received from the data controller via private communication, and so should be reasonably accurate.

- There is often discussion of linked datasets out of context of the core dataset which I think adds to the confusion. For example, lines 63-4 reference "two standalone national ICU audit datasets and a linked dataset." However, line 104 describes linked datasets in a way that seems to combine all three Is this an analytic dataset that is being referenced?

Response:

Sorry for the confusion. The linked dataset is based on core data (HES Critical Care) linked to the two standalone ICU datasets and other hospital use data. We have rephrased the description in the revised manuscript to explain it more clearly (line 56-60):

"*There will be three datasets: two standalone national ICU audit datasets and a linked dataset (which combines the core critical care data with the standalone datasets, hospital use data, and survival status). Table 1 provides descriptions of all data sources, all of which are routinely collected clinical audit or national datasets, and the inclusion and exclusion criteria used to select patients from these datasets.* "

- Additionally, restructuring the analysis plans may be useful, to the above

point. Having the standalone first, followed by the “linked dataset” or vice versa, though remaining consistent in how they were introduced in lines 66-89.

Response:

We appreciate your suggestion. In the revised manuscript, we have restructured the data introduction by describing the core dataset (and linked dataset) first, followed by the two standalone datasets. This order now aligns with the data analysis plan.

- Lines 153-154 suggest missing data will be inferred from different sources. Which different sources?

Response:

We meant that in the linked dataset, we will use data from different sources to improve data completeness. In the revised version, we have rephrased the sentence to make this clearer (line 151).

“In the linked dataset, we will use data from different sources to enhance data completeness.”

- It is novel to include non-English speaking individuals; however, can you go into additional detail on how they will be consented? The methods discuss translators during interviews, though will short forms and/or translated consents be used? Are all of those languages available for consent? Also will translation be done by a person (limited language capabilities) or a virtual service (i.e. language line)?

Response:

A more detailed explanation of the consent and translation procedure has been provided within the manuscript to offer more clarity (line 202 – 222).

- Line 221 describes using “the seven-stage process of transcription.” Yet there is no citation to the methods for this transcription or the qualitative methodology which is mentioned later, “Framework” and thematic analysis.

Response:

Thank you for highlighting the missing reference and awkwardly written sentence. The seven-stages refers to the analysis process involved in Gale et al’s Framework Method, not seven-stages of transcription. Transcription is the first stage of the process. This was not clear in the original sentence and this has been amended in the manuscript to offer more clarity (line 229–238):

“Topic guides will be iteratively refined based on early interviews to ensure alignment with participant perspectives. Demographic data will be collected to contextualise findings. All interviews will be audio recorded and transcribed by an independent translation service. Where a translator is required, the audio recording will be transcribed in both English and the language used by the participant and interpreter. Data from interviews will be analysed using the Framework Method, following Gale et al.’s seven-stages of analysis; transcription, familiarisation, coding, development of a working analytical framework, indexing, charting and interpreting the data. Data

sufficiency will be assessed through ongoing team discussion and be determine by consensus when thematic saturation has been reached in relation to the research question."

Additionally, updated references have been included to support the use of Braun and Clarke's reflexive thematic analysis (line 278) along with additional explanation for the use (line 278-281).

- Individuals will be eligible for inclusion based on demographic information during registration. How will the authors ensure that individuals are actually within the target population of interest? (i.e. not an impersonator causing nonsensical responses)

<u>Response:</u>

We appreciate the reviewer's concern regarding the potential for inauthentic participation during the online forum. While individuals will be asked to provide basic demographic information and respond to brief screening questions upon registration, we acknowledge that it is not possible to verify identity with absolute certainty in an online context. However, the online discussion will be monitored closely, and any responses that appear nonsensical, irrelevant, or inconsistent with the study aims will be reviewed and, if necessary, removed from the dataset.

The manuscript has been amended to include clarity of this process (line 254-261):

"Interested participants will be directed to closed Facebook groups, where they will be asked to provide basic demographic information and respond to brief screening questions during registration for eligibility verification; however full verification of identify will not be possible in an online context. Upon confirmation, participants will be able to join the asynchronous forums and contribute to discussions. Participation will be voluntary, and engagement with the forum will imply consent. Forum contributions will be monitored closely, and any responses that appear inauthentic or inconsistent with the study aims will be reviewed and, if necessary, removed."

Patient and Public Involvement
- I commend the authors on inclusion of the PPI in study design. Is there compensation for this research? It seems that a considerable amount of time will be taken to participate in focus groups.

<u>Response:</u>

Participants involved as part of the PPIE group will be reimbursed for their time in line with national guidelines, in recognition of their valuable contribution to the research. Funding has been allocated specifically to ensure appropriate compensation for their involvement.

A sentence confirming this has been added to the manuscript to offer further clarification (line 373-375):

"Participants involved in the group will be reimbursed for their time in line with national guidance, in recognition of the valuable contribution they make to the research."

- There is mention of parent co-applicant. Can you expand on this? Is this for funding of this project, and if so, is it funded?

Response:

The parent co-applicant has a funded and defined role within the research team, including reviewing the grant application and study materials, contributing to data analysis and leading the PPIE group. More information concerning this role has been added to the revised manuscript to offer more clarity (line 366-368):

"The PPI co-applicant has a defined and funded role within the research team, including reviewing the grant application and study materials, providing input on data collection methods and interpretation as well as leading the PPIE group."

Figures:
- Figure 1 is a bit confusing and doesn't answer as to why the standalone datasets are needed, and what exactly they are adding to the core dataset. Perhaps more in-depth explanation in the text, and figure legends may assist as figures should be able to stand on their own.

Response:

In the revised version, we have highlighted the advantages of standalone dataset in line 71-73:

"It will also be linked to records between 2017-2023 in the two standalone ICU datasets (see details below) for those had admission aged 14-15 as these contain much more detailed data on reasons for ICU admission and support provided"

We also added one sentence in the Figure 1 legend to explain the importance of including them:

"The standalone datasets (PICANet and ICNARC CMP) cover a longer time period than HES CC and include more detailed clinical information, enabling analysis of ICU usage trends over time and patient pathways."

- Figure 2 outlines the trichotomous outcomes of ICU use in "Output." This needs to be expanded upon and described. What is meant by patterns, and what are the 3 types?

Response:

Figure 2 is illustrative, and we do not yet know how many distinct phenotypes will emerge among patients who required ICU care after age 16. The "patterns" will be related to frequency of admission, length of stay, and whether the patient was admitted to the PICU or AICU, etc. We have revised Figure 2 to clarify this.

Strengths and limitations
- There is little discussion regarding limitations besides small sample size. It is difficult to ascertain the true proposed sample size. Please specify.

Response:

The estimates of the number of patients admitted at ages 14–15 were obtained from the data controller via private communication. However, the sample size of those who experienced a transition (i.e., had another ICU admission after age 16) is difficult for anyone to estimate, as this is a highly understudied population. In the revised version, we have added the discussion under Section "Main cohort and comparator cohort" (line 98-102):

"The sample size of the main cohort is difficult to estimate because this is an understudied population, and determining this number is one of the key aims of OPTICAL. Our study is also limited by a potentially small sample size, given the relatively short time frame of the linked dataset (2017–2024). However, within this period, we will have access to the entire national cohort, providing the largest possible sample available for this population."

- An additional limitation would be the inability to fully link the datasets into an analytic dataset due to differing variables.
Response:

The linkage will be done by matching patient identifiers (see Figure 1). The availability of different variables from multiple data sources will enable a more detailed analysis of ICU use and hospital resource utilization; therefore, this is not considered a limitation.

Reviewer: 2
Dr. Haiying Wu, The First Affiliated Hospital of Kunming Medical University

Comments to the Author:
1.Clarify Integration Strategy Across Work Packages
Please elaborate more clearly on how findings from WP1 (quantitative data), WP2 (qualitative interviews and forums), and WP3 (stakeholder engagement) will be integrated. Consider including a schematic or paragraph detailing your planned triangulation or synthesis strategy.

Response:

Thank you for your valuable suggestion. Work package 3 is an overarching strand of the project focused on integrating the findings from WPs 1 and 2 within a structured stakeholder engagement process to establish evidence-based improvements in transition services. The integration strategy in WP3 follows the approach of Crowe et al. 2016, which outlines how the findings from multiple sources of qualitative data were used in combination with quantitative data to inform the development of recommendations to improve services for infants with congenital heart disease following discharge from hospital [Crowe et al. BMJ Open 2016;6:e010363]. Within OPTICAL, this approach includes: synthesising the findings from the different components of WP2 (interviews, forums, surveys) using a hyper-framework to identify archetypal service problems and candidate service improvements that address them; the formation of an expert stakeholder group to consider, refine and

prioritise these candidate service improvements through a structured and systematic engagement process; consideration of the quantitative findings (WP1) within the refinement and prioritisation of improvements, in particular whether any should be targeted at patients with particular characteristics given limited resources available to services.

The bottom part of Figure 1 presents a schematic of the work package flows. Note that the expert group convened as part of WP3 will also review and potentially inform aspects of work packages 1 and 2 (e.g. they may suggest some changes to survey questions, based on their review of the emerging findings from the interviews and forums in WP2), hence the arrows in Figure 1 flow in both directions between WP1 and WP3, and between WP2 and WP3.

In the revised manuscript, we have added a paragraph (line 308-314) to explain the integration strategy across work packages.

2.Define Technical Terms
Some methodological terms such as "hyper-framework" and "problem structuring methods" may be unfamiliar to a broad medical audience. We recommend briefly defining these upon first use to improve clarity and accessibility.

Response:

Thank you for pointing this out. In the revised manuscript, we have added more explanations for methodological terms. For example, the terms 'hyper-framework' and 'hyper-theme' are explained in more detail in lines 326–330, and 'problem structuring method' is explained in lines 339–341.

3.Address Potential Sample Size Limitations
Although the study acknowledges a limited number of transition cases in the linked dataset (2017–2023), a more detailed discussion of how this may impact generalisability and statistical power would strengthen the protocol.

Response:

In the revised manuscript, we have expanded the discussion of the limited number of transition cases in the linked dataset in line 98-102:

"The sample size of the main cohort is difficult to estimate because this is an understudied population, and determining this number is one of the key aims of OPTICAL. Our study is also limited by a potentially small sample size, given the relatively short time frame of the linked dataset (2017–2024). However, within this period, we will have access to the entire national cohort, providing the largest possible sample available for this population."

Our study is a cohort study based on nationally routinely collected healthcare data. The study will be largely descriptive and we are not testing a hypothesis in this research. Therefore, a discussion of sample size and statistical power is not included.

4.Provide Saturation Strategy for Qualitative Components

While you plan 120 interviews and 200 forum participants, please indicate how thematic saturation will be assessed and whether you anticipate iterative refinement based on early data.

<u>Response:</u>

Topic guides will be iteratively refined based on early interview data, as noted in the Data Collection and Analysis section (*"Topic guides will be iteratively refined on early interviews to ensure alignment with participant perspectives").* For the interview, data sufficiency will be assessed through ongoing team discussion, and we will determine by consensus when thematic saturation has been reached in relation to the research question (line 236-238 in the revised manuscript):

"Data sufficiency will be assessed through ongoing team discussion and be determine by consensus when thematic saturation has been reached in relation to the research question."

The online forum will run for a fixed period and be actively moderated; while saturation may not be formally assessed, new prompts will be introduced pragmatically when engagement with a topic diminishes. Additional clarification has been added into the manuscript (line 275-276):

"While data saturation will not be formally assessed, new prompts will be introduced pragmatically when engagement with a topic diminishes".

5.Refer to Reporting Guidelines Explicitly
Consider including a brief note stating that the protocol follows relevant reporting standards (e.g., STROBE, SPIRIT, or GRAMMS for mixed-methods studies), and ensure that the checklist is submitted as supplementary material.

<u>Response:</u>

This is a cohort study, so results regarding WP1 will be reported in accordance with the STROBE guidelines (as clarified in the revised manuscript, line 392). However, as this paper focuses on the study protocol and the core data have not yet been received/analysed, the reporting checklist cannot be completed at this stage and therefore will not be submitted at this time.

6.Consider Streamlining Background Section
The introduction is comprehensive but somewhat lengthy. Consider condensing the background by summarising widely known guidelines or prior studies into fewer lines to improve readability.

<u>Response:</u>

Thank you for your suggestion. In the revised manuscript, we have trimmed the introduction to improve readability.
